# Could daily changes in respiratory microbiota help predicting early *Staphylococcus aureus* ventilator-associated pneumonia?

**DOI:** 10.1186/s40635-023-00521-7

**Published:** 2023-06-23

**Authors:** Sylvain Meyer, Nadia Gaïa, Vladimir Lazarevic, Jacques Schrenzel, Bruno François, Olivier Barraud, the SCORPIUS study group, Thomas Daix, Delphine Chainier, Marie-Cécile Ploy, Philippe Vignon

**Affiliations:** 1 Université Limoges, INSERM, CHU Limoges, UMR 1092, Limoges, France; 2 Genomic Research Laboratory and Infectious Diseases Division, Geneva University Hospitals and University of Geneva, Geneva, Switzerland; 3 CHU Limoges, Réanimation Polyvalente CHU Dupuytren, 2 Ave. Martin Luther King, 87042 Limoges Cedex, France; 4 CHU Limoges, INSERM, CIC1435 Limoges, France

**Keywords:** Ventilator-associated pneumonia, Respiratory microbiota, *Staphylococcus aureus*, Anaerobes

Dear Editor,

Ventilator-associated pneumonia (VAP) remains the most frequent healthcare-associated infection in Intensive Care Units (ICUs) with a prevalence of 9–27% [1]. Factors driving the progression from colonization to infection during VAP development are not fully elucidated. Metataxonomics [16S rRNA gene next-generation sequencing (NGS)] has emerged as an efficient tool to investigate pulmonary microbiota. When compared to conventional aerobic culture, metataxonomics provides additional information which allows assessing exhaustively the local bacterial community, including anaerobes, that are fastidious and yet-unculturable organisms. This culture-free approach provides new insights into respiratory microbiota dynamics and potential mechanisms of VAP development [2–5]. No study has yet sequentially explored respiratory microbiota dysbiosis on a daily basis and its potential relationship with the development of early VAP. We hypothesized post hoc that patients who develop *Staphylococcus aureus* VAP might exhibit modified respiratory microbiota during mechanical ventilation (MV) when compared to patients without VAP. In this pilot study (IRB #464-2021-120), we analyzed the daily changes of respiratory microbiota in a homogeneous population of patients under MV without prior exposure to antibiotics or antibiotic treatment during the study period until potential VAP diagnosis.

Eligible patients were adults admitted to the ICU for an acute brain injury requiring at least 48 h of MV, with no chronic or acute respiratory disease, and who did not receive antibiotics in the previous 15 days. If antibiotics were administered during the study period, patients were secondarily excluded. Endotracheal aspirates (ETA) were collected every morning from the day of tracheal intubation (day 1) to either the day of VAP suspicion, the day of extubation or until day 7 whichever occurred first. All VAP events were blindly adjudicated by two independent ICU physicians.

Twelve patients were included (Additional file 1: Table S1) with a total of 62 ETA samples. Four patients developed *S. aureus* VAP (3 early- and 1 late-onset) (Additional file 1: Table S2) and 8, including 4 colonized with *S. aureus*, were considered non-VAP controls. Clinical characteristics at baseline were comparable between groups (Additional file 1: Table S1). Microbiota analysis (Additional file 1: Table S3) consistently confirmed the presence of bacterial species identified by culture even if their relative abundance was not related to CFU counts (Additional file 1: Figure S1). Incidence of *S. aureus* was high because of study population and local epidemiology. On day 1, the differences in microbiota between patients who will develop VAP and controls were not statistically significant, and microbiota evolved over time in both groups (Fig. 1, Additional file 1: Figure S2). The alpha-diversity shifts during follow-up showed no common trends in VAP or non-VAP patients. However, we were able to identify specific bacterial taxa with relative abundances significantly higher in early *S. aureus* VAP than in non-VAP patients (e.g., *Prevotella* species, *Neisseria flavescens*) (Additional file 1: Table S4). These taxa possibly provide a metabolic niche for *S. aureus*, as suggested in the context of a different infection [6]. They could constitute potential PCR targets for future clinical ICU practice. Canonical analysis of principal coordinates showed microbial profiles that could also potentially be used as predictors of early *S. aureus* VAP (Additional file 1: Figure S3). This underlines the potential benefit of microbiota analysis over other diagnostic tools, such as targeted PCRs, to predict *S. aureus* VAP.

**Fig. 1:**
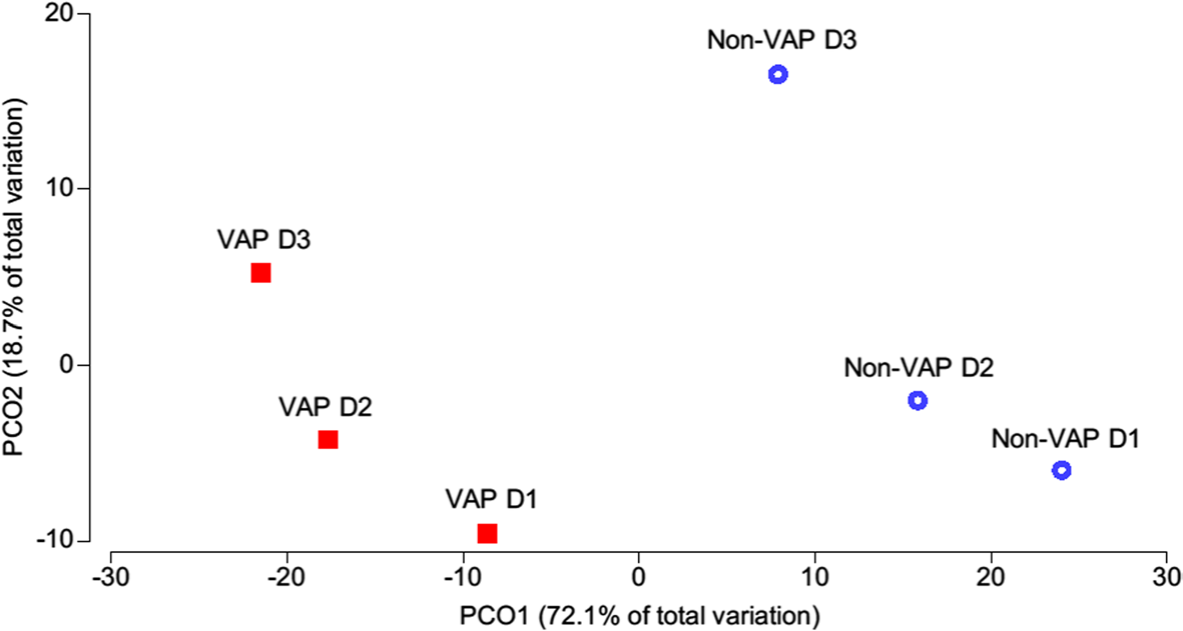
Microbiota differences of early *S. aureus* VAP vs non-VAP patients during the first 3 days of intubation assessed by Principal coordinates analysis (PCoA). PCoA was based on the Bray–Curtis dissimilarity matrix of square-root transformed relative abundances of bacterial species. Bacterial communities defined by the sampling day and VAP occurrence were grouped to centroids. For this analysis of centroids, patient 1 was considered “non-VAP” as the VAP occurred on day 7 (late VAP). Difference was not significant on day 1 (PERMANOVA *p* > 0.05). D = day post-intubation

This proof-of-concept study allowed to identify respiratory microbiota changes during the first days of ICU admission that preceded the development of *S. aureus* VAP. Whether microbiota may be predictive of subsequent development of VAP remains to be tested in other homogeneous ICU populations to confirm these preliminary results.

## Supplementary Information

**Additional file 1. Table S1.** Patients characteristics at ICU admission. **Table S2.** Radiologic and biologic characteristics of VAP patients the two days before VAP diagnosis. **Table S3.** Reagent contaminant species. **Table S4.** Differences in microbiota composition between early S. aureus VAP and non-VAP groups at different taxonomic levels at Day 1 and Day 2. **Figure S1.** Daily evolution of main bacterial genera of the respiratory microbiota for each patient enrolled in the study. **Figure S2.** Microbiota similarities/differences assessed by Principal coordinates analysis. **Figure S3.** Receiver operating characteristiccurve for associations between an early S. aureus VAP and bacterial communities at Day 1 and Day 2 of intubation. Supplementary Methods and Results.

## Data Availability

The data sets generated for this study can be found in the Sequence Read Archive (SRA), PRJNA882222.
