# Supplementary material for: Could daily changes in respiratory microbiota help predicting early Staphylococcus aureus ventilator-associated pneumonia?
Source: Intensive Care Med Exp. 2023 Jun 23;11:34. doi: 10.1186/s40635-023-00521-7 (PMC10287595; doi:10.1186/s40635-023-00521-7)
Supplement: Supplementary file 1 — Additional file 1. Table S1. Patients characteristics at ICU admission. Table S2. Radiologic and biologic characteristics of VAP patients the two days before VAP diagnosis. Table S3. Reagent contaminant species. Table S4. Differences in microbiota composition between early S. aureus VAP and non-VAP groups at different taxonomic levels at Day 1 and Day 2. Figure S1. Daily evolution of main bacterial genera of the respiratory microbiota for each patient enrolled in the study. Figure S2. Microbiota similarities/differences assessed by Principal coordinates analysis. Figure S3. Receiver operating characteristiccurve for associations between an early S. aureus VAP and bacterial communities at Day 1 and Day 2 of intubation. Supplementary Methods and Results. [file 40635_2023_521_MOESM1_ESM.docx]

**Could daily changes in respiratory microbiota help predicting early *Staphylococcus aureus* ventilator-associated pneumonia?**

Sylvain Meyer, Nadia Gaïa, Vladimir Lazarevic, Jacques Schrenzel, Bruno François and Olivier Barraud on behalf of the SCORPIUS study group

**Supplementary Material**

**Table S1: Patients characteristics at ICU admission.**

|  | **Total**  (n=12) | **Non-VAP**  (n=8) | **VAP**  (n=4) |
| --- | --- | --- | --- |
| **Sex M/W** | 9 (75%) / 3 (25%) | 5 (63%) / 3 (37%) | 4 (100%) / 0 (0%) |
| **Age** | 65.5 [63.8 – 70.3] | 62.5 [59.8 – 70.3] | 68.8 [65.8 – 71.0] |
| **Admission** |  |  |  |
| Traumatic brain injury | 2 (17%) | 1 (13%) | 1 (25%) |
| Hemorrhagic stroke | 6 (50%) | 4 (50%) | 2 (50%) |
| Subarachnoidal hemorrhage | 2 (17%) | 1 (13%) | 1 (25%) |
| Insulin intoxication | 2 (17%) | 2 (25%) | 0 |
| **Prehospital intubation** | 5 (42%) | 3 (38%) | 2 (50%) |
| **Charlson index** | 3.3 [2.0 – 4.3] | 3.5 [1.8 – 5.3] | 3.0 [2.8 – 3.3] |
| **SAPSII** | 52 [46 – 59] | 52 [47 – 59] | 52 [46 – 55] |
| **Glasgow Coma Score** | 7 [4 – 9] | 7 [4 – 9] | 8 [7 – 9] |

Qualitative variables are expressed in n (%) and quantitative continuous variables in median [Q1 – Q3].

M: men; W: women; VAP: Ventilator-Associated Pneumonia; SAPSII: Simplified Acute Physiology Score II

**Table S2: Radiologic and biologic characteristics of VAP patients the two days before VAP diagnosis**

|  | Patient 1 | | | Patient 2 | | | Patient 6 | | | Patient 11 | | |
| --- | --- | --- | --- | --- | --- | --- | --- | --- | --- | --- | --- | --- |
|  | Day VAP-2 | Day VAP-1 | Day VAP | Day VAP-2 | Day VAP-1 | Day VAP | Day VAP-2 | Day VAP-1 | Day VAP | Day VAP-2 | Day VAP-1 | Day VAP |
| Chest X-ray7 | No infiltrate | NA | Localized | NA | Localized | Localized | Diffused | Diffused | Localized | Diffused | NA | Localized |
| Temperature (°C) | 37.6 | 38.7 | 38.9 | 38.4 | 39.4 | 39.1 | 39.1 | 39.6 | 38.9 | 37.6 | 38.2 | 38.5 |
| Leukocytes (G/L) | 6.63 | 7.67 | 8.95 | 13.32 | NA | 10.10 | 8.69 | 10.57 | 10.80 | 15.34 | 1.73 | 0.98 |
| PaO2/FiO2 (mmHg) | 383 | NA | 458 | 226 | 273 | 189 | 523 | 202 | 294 | 208 | 187 | 235 |
| CPIS | 3 | 4 | 5 | 5 | 6 | 8 | 5 | 7 | 5 | 6 | 6 | 8 |

Day VAP-2: two days before VAP diagnosis; Day VAP-1: one day before VAP diagnosis

CPIS: Clinical Pulmonary Infection Score. NA: data not available

**Table S3. Reagent contaminant species.** Species whose read counts in control samples were higher than those of 62 ETA samples were considered contaminants.

| **Species** | **Average read counts in negative controls *** | **Average read counts in ETA samples *** | **Putative contaminant** |
| --- | --- | --- | --- |
| *Microbacterium ginsengisoli* | 3.8 | 0 | Yes |
| *Bradyrhizobiaceae*_unclassified | 10 | 0 | Yes |
| *Cupriavidus*_unclassified | 6.2 | 0 | Yes |
| *Ralstonia insidiosa* | 14 | 0 | Yes |
| *Ralstonia pickettii* | 1.0 | 0 | Yes |
| *Pelomonas puraquae* | 22 | 0 | Yes |
| *Marmoricola_*unclassified | 1.2 | 0 | Yes |
| *Staphylococcus*_unclassified | 2.5 | 9816 | No |
| *Moraxellaceae*_unclassified | 0.8 | 1.4 | No |
| *Solobacterium*_unclassified | 1.0 | 2178 | No |
| *Prevotella*_unclassified | 1.5 | 12982 | No |

* normalized per million of classified reads

**Table S4: Differences in microbiota composition between early *S. aureus* VAP and non-VAP groups at different taxonomic levels at Day 1 and Day2.**

|  | Day 1 | | | | | | | Day 2 | | | | | | |
| --- | --- | --- | --- | --- | --- | --- | --- | --- | --- | --- | --- | --- | --- | --- |
|  | Relative abundance* | | Number of samples positive for a given taxon | |  | |  | Relative abundance* | | Number of samples positive for a given taxon | |  | |  |
| Taxonomy | VAP | Non-VAP | VAP | Non-VAP | p-value** | adjusted p-value | | VAP | Non-VAP | VAP | Non-VAP | p-value** | adjusted p-value | |
| **Phylum** |  |  |  |  |  |  | |  |  |  |  |  |  | |
| Bacteroidetes | 17.25 | 1.76 | 3 | 9 | 0.018 | 0.073 (NS) | | 19.65 | 5.76 | 3 | 8 | 0.064 (NS) | 0.255 (NS) | |
| **Class** |  |  |  |  |  |  | |  |  |  |  |  |  | |
| Bacteroidia | 17.24 | 1.76 | 3 | 9 | 0.018 | 0.145 (NS) | | 19.54 | 5.75 | 3 | 8 | 0.064 (NS) | 0.361 (NS) | |
| **Order** |  |  |  |  |  |  | |  |  |  |  |  |  | |
| Bacteroidales | 17.24 | 1.76 | 3 | 9 | 0.018 | 0.182 (NS) | | 19.54 | 5.75 | 3 | 8 | 0.064 (NS) | 0.159 (NS) | |
| Pasteurellales | 0.34 | 0 | 3 | 4 | 0.125 (NS) | 0.313 (NS) | | 0.33 | 0 | 3 | 4 | 0.013 | 0.127 (NS) | |
| **Family** |  |  |  |  |  |  | |  |  |  |  |  |  | |
| Pasteurellaceae | 0.34 | 0 | 3 | 4 | 0.125 (NS) | 0.438 (NS) | | 0.33 | 0 | 3 | 4 | 0.013 | 0.266 (NS) | |
| Porphyromonadaceae | 2.74 | 0.09 | 3 | 8 | 0.209 (NS) | 0.488 (NS) | | 4.69 | 0.82 | 3 | 8 | 0.036 | 0.267 (NS) | |
| Prevotellaceae | 9.07 | 1.67 | 3 | 8 | 0.018 | 0.382 (NS) | | 15.00 | 3.57 | 3 | 8 | 0.064 (NS) | 0.267 (NS) | |
| **Genus** |  |  |  |  |  |  | |  |  |  |  |  |  | |
| *Catonella* | 0.31 | 0 | 3 | 4 | 0.013 | 0.176 (NS) | | 0.40 | 0 | 3 | 3 | 0.048 | 0.308 (NS) | |
| *Haemophilus* | 0.24 | 0 | 3 | 4 | 0.125 (NS) | 0.403 (NS) | | 0.07 | 0 | 3 | 4 | 0.035 | 0.308 (NS) | |
| *Howardella* | 0.03 | 0 | 3 | 2 | 0.039 | 0.284 (NS) | | 0.01 | 0.02 | 3 | 5 | 0.851 (NS) | 0.884 (NS) | |
| *Pasteurellaceae_unclassified* | 0.10 | 0 | 3 | 1 | 0.016 | 0.176 (NS) | | 0.08 | 0 | 3 | 0 | 0.002 | 0.047 | |
| *Prevotella* | 8.81 | 1.67 | 3 | 8 | 0.018 | 0.176 (NS) | | 13.42 | 3.00 | 3 | 8 | 0.064 (NS) | 0.308 (NS) | |
| **Species** |  |  |  |  |  |  | |  |  |  |  |  |  | |
| *Catonella morbi* | 0.11 | 0 | 3 | 4 | 0.013 | 0.264 (NS) | | 0.36 | 0 | 3 | 3 | 0.048 | 0.532 (NS) | |
| *Howardella_unclassified* | 0.03 | 0 | 3 | 2 | 0.040 | 0.352 (NS) | | 0.01 | 0.02 | 3 | 5 | 0.851 (NS) | 0.926 (NS) | |
| *Neisseria flavescens* | 0.05 | 0 | 3 | 1 | 0.004 | 0.216 (NS) | | 0.22 | 0 | 3 | 0 | 0.002 | 0.041 | |
| *Pasteurellaceae_unclassified* | 0.10 | 0 | 3 | 1 | 0.016 | 0.264 (NS) | | 0.08 | 0 | 3 | 0 | 0.002 | 0.041 | |
| *Prevotella maculosa* | 0.35 | 0 | 3 | 3 | 0.048 | 0.352 (NS) | | 0.55 | 0 | 3 | 4 | 0.055 (NS) | 0.532 (NS) | |
| *Prevotella melaninogenica* | 2.68 | 0.10 | 3 | 6 | 0.041 | 0.352 (NS) | | 6.69 | 0.18 | 3 | 6 | 0.063 (NS) | 0.532 (NS) | |

p-values highlighted in orange are showing a statistically-significant increase in VAP relative to non-VAP patients before correction for multiple testing with Benjamini-Hochberg method. * Median value of the percentage of reads assigned to a given taxon. ** Mann-Whitney-Wilcoxon test. NS, not significant (p > 0.05). In this analysis, patient 1 was considered “non-VAP” as the VAP occurred at Day 7 (late VAP). VAP: Ventilator-Associated Pneumonia.

**Figure S1: Daily evolution of main bacterial genera of the respiratory microbiota for each patient enrolled in the study.**

Top graph in each panel: Relative abundances of main bacterial genera (family for Enterobacteriaceae) during the first seven days of intubation (or less if the patient developed VAP or was extubated) as assessed by metataxonomics. Black dots represent CPIS score. Red arrows indicate the day of VAP diagnosis for patients 1, 2, 6 and 11.

Bottom graph in each panel: Quantitative culture results (expressed in CFU/ml) with potential pathogens identified.

CPIS: Clinical Pulmonary Infection Score; CFU: Colony Forming Unit.

**Figure S2: Microbiota similarities/differences assessed by Principal coordinates analysis (PCoA).**

A. Daily evolution of the microbiota can be visualized for each patient.

B. Samples differentiated by development of VAP and sampling point (D1–D4). D1–D3 samples were available for each patient whereas samples from following days (D4–D7) were not always available and were combined into “D4+”.

C. Bacterial communities defined by the sampling day and VAP occurrence were grouped to centroids. Difference was not significant at Day 1 (PERMANOVA p > 0.05). Vectors of Pearson correlation (> 0.7) between species relative abundance and PCo axes are shown at the PCoA origin.

ETA: endotracheal aspirate; D: day post intubation; VAP: ventilator-associated pneumonia

**Figure S3: Receiver operating characteristic (ROC) curve for associations between an early *S. aureus* VAP and bacterial communities at Day 1 and Day 2 of intubation.**

Sample were allocated to VAP or non-VAP group by the Canonical Analysis of Principal coordinates (CAP) following the cross-validation procedure. The CAP is a constrained ordination method that maximizes separation of pre-defined groups (VAP and non-VAP) in the multivariate space, based on their (bacterial) community profiles. This method calculates the proportion of successful allocations to these two groups and can also be used for future predictions i.e. allocation of new samples, given the resemblance of their (bacterial) communities with existing VAP and non-VAP samples. Patient 1 with VAP-onset at Day 7 (late VAP) was considered “non-VAP”.

At Day 1 after intubation (in blue), respiratory microbiota composition between both groups showed a sensitivity and a specificity of 33.3% and 88.9%, respectively. At Day 2 after intubation (in red), these values reached 100%. Therefore, CAP enabled to discriminate patients developing VAP based on respiratory microbiota composition.

**Supplementary Material:**

*VAP diagnosis*

VAP diagnosis was based on Food and Drug Administration guidance for diagnosis and confirmation of VAP criteria from 2010, and was retrospectively adjudicated by two independent ICU physicians after careful analysis of clinical, biological, radiological and microbiological data (Table S2). All patients with VAP had a confirmed localized infiltrate in chest X-ray the day of VAP diagnosis. Pneumonia that occurred 3–5 days after intubation was considered early-onset VAP. Patients with obvious or suspected aspiration were excluded from inclusion based on the medical history and the report of the intubation.

*Human DNA depletion and 16S rRNA gene sequencing*

Five hundred microliters of raw ETA samples were liquefied with 500 µL of Digest-EUR® (Eurobio) in a sterile tube. Human DNA was depleted from liquefied ETA using the MolYsis Basic5 Kit (Molzym) as recommended by the manufacturer. Bacterial DNA was extracted with the Bacterial DNA Extraction Kit on the SaMag-12® automatic system (Sacace Biotechnologies). For each depletion/extraction series, a negative control consisting of 500 µL of sterile water was included and followed the same protocol as ETA samples in order to reveal potential kit contaminants. The 16S rRNA gene was amplified using the Ion 16S™ Metagenomics Kit (Life Technologies) which enables the amplification of seven of the nine variable regions of the 16S rRNA gene. PCR products were purified using Agencourt AMPure XP beads (Beckman Coulter) and then barcoded using the Ion Fragment Plus Core Library Kit on AB Library Builder™ System (Life Technologies). Amplicons were sequenced on the Ion GeneStudio S5™ Platform (Life Technologies) using a 530 chip (400 bp single-end) according to manufacturer’s instructions. Twenty ETA samples were sequenced per chip.

*Bioinformatics*

Raw BAM files were uploaded to the Ion Reporter v5.16 platform (ThermoFisher Scientific) to generate species abundance profiles against the MicroSEQ™ ID 16S rDNA 500 Library v2013.1 database using a confidence threshold > 99%. A minimum threshold of 10 reads per species was defined in Ion Reporter platform. *Ralstonia, Bradyrhizobium, Cupriavidus, Pelomonas* and *Microbacterium* genera were usual contaminants found in negative controls (Table S4) and were excluded from sample analysis. Rarefaction curves were generated for each sample and for each variable region using vegan R package v2.5-6.

Alpha-diversity (Shannon index and species richness) were computed using the PRIMER v7.0.21 (PRIMER-e) software. Beta-diversity (Principal Coordinates Analysis, PCoA), PERMANOVA test and Canonical Analysis of Principal coordinates (CAP) were performed with PRIMER using Bray-Curtis dissimilarity matrix based on square-root transformed taxa relative abundances.

To assess differences in the relative abundance of bacterial taxa between VAP and non-VAP patients, we used Mann-Whitney-Wilcoxon test. An uncorrected p-value *<*0.05 was considered statistically significant.

*16S rRNA sequencing results*

An average number of 322 418 reads per sample was obtained after filtering and taxonomic assignment with regions V3, V8, V6-V7 and V4 being the most frequently sequenced. Rarefaction curves showed that species diversity was fully captured for all sequenced regions. The highest number of species was identified in the V3 region (n=213), and this region provided the highest average number of species per ETA sample. Therefore, we decided to conduct further analyses using the V3 region sequence data. Partial sequencing of the 16S rRNA gene limits species identification. Nonetheless, the choice of the V3 region allowed identification at the species level for 122 (57.3%) out of the 213 taxa (others were labeled as “genus_unclassified” or “family_unclassified”).
